# Genotyping 1000 yeast strains by next-generation sequencing

**DOI:** 10.1186/1471-2164-14-90

**Published:** 2013-02-09

**Authors:** Stefan Wilkening, Manu M Tekkedil, Gen Lin, Emilie S Fritsch, Wu Wei, Julien Gagneur, David W Lazinski, Andrew Camilli, Lars M Steinmetz

**Affiliations:** 1 Genome Biology Unit, European Molecular Biology Laboratory, Meyerhofstr. 1, 69117, Heidelberg, Germany; 2 Gene Center Munich, Department of Chemistry and Biochemistry, Ludwig-Maximilians-Universität München, Feodor-Lynen-Str. 25, 81377, Munich, Germany; 3 Department of Molecular Biology & Microbiology and Howard Hughes Medical Institute, Tufts University, 136 Harrison Avenue, Boston, MA, 02111-1817, USA

**Keywords:** Next-generation sequencing, High throughput, DNA isolation, Yeast, DNA fragmentation, Heat inactivation, Recombination, Aneuploidy

## Abstract

**Background:**

The throughput of next-generation sequencing machines has increased dramatically over the last few years; yet the cost and time for library preparation have not changed proportionally, thus representing the main bottleneck for sequencing large numbers of samples. Here we present an economical, high-throughput library preparation method for the Illumina platform, comprising a 96-well based method for DNA isolation for yeast cells, a low-cost DNA shearing alternative, and adapter ligation using heat inactivation of enzymes instead of bead cleanups.

**Results:**

Up to 384 whole-genome libraries can be prepared from yeast cells in one week using this method, for less than 15 euros per sample. We demonstrate the robustness of this protocol by sequencing over 1000 yeast genomes at ~30x coverage. The sequence information from 768 yeast segregants derived from two divergent *S. cerevisiae* strains was used to generate a meiotic recombination map at unprecedented resolution. Comparisons to other datasets indicate a high conservation of recombination at a chromosome-wide scale, but differences at the local scale. Additionally, we detected a high degree of aneuploidy (3.6%) by examining the sequencing coverage in these segregants. Differences in allele frequency allowed us to attribute instances of aneuploidy to gains of chromosomes during meiosis or mitosis, both of which showed a strong tendency to missegregate specific chromosomes.

**Conclusions:**

Here we present a high throughput workflow to sequence genomes of large number of yeast strains at a low price. We have used this workflow to obtain recombination and aneuploidy data from hundreds of segregants, which can serve as a foundation for future studies of linkage, recombination, and chromosomal aberrations in yeast and higher eukaryotes.

## Background

**Figure 1:**
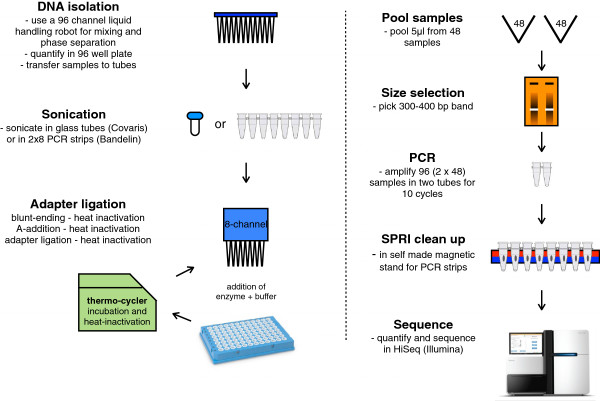
**Library preparation pipeline.** DNA isolation is performed with a 96-head liquid handling robot. DNA fragmentation is achieved by sonication, either in glass tubes (Covaris) or PCR strips (Bandelin). SPRI bead cleanup is automated on a 96-head liquid handling robot. Three enzymatic steps for barcoded adapter ligation are performed by addition of enzyme (+ buffer), incubation, and heat inactivation in a thermocycler. After pooling of 48 barcoded libraries, samples are concentrated and size-selected using an E-gel. PCR is performed on the size-selected pool to enrich for adapter containing fragments and elongate them to full-length libraries. A final cleanup is performed in PCR strips mounted to a homemade magnetic stand.

The increase in throughput of next-generation sequencing (NGS) machines has enabled the use of whole-genome or targeted sequencing for biological and clinical studies at an unprecedented scale [1]. Despite the decrease in the price of sequencing itself, the cost and time for preparation of sequencing libraries limit the affordability and feasibility of sequencing large numbers of genomes. Various DNA sequencing protocols have been developed to increase the throughput and decrease the price per sample preparation [2-11] (for a summary of published protocols, see Additional file 1: Table S1). The sample preparation pipeline (Figure 1) that we present here consists of a DNA isolation method from yeast cells performed in 96-well plates yielding high-quality genomic DNA, a DNA fragmentation method performed in PCR tubes with a sonicating water bath, and a heat inactivation step to circumvent the cleanups. Depending on individual requirements, individual steps of our workflow can be integrated into other workflows. We applied this pipeline to *S. cerevisiae*, a model organism of choice for genetic and genomic studies [12-15]. Using this pipeline, we sequenced over 1000 yeast genomes, including 768 meiotic segregants from a cross between two distantly related *S. cerevisiae* strains [16], namely haploid derivatives of S288c (i.e., S96) and SK1. The quality of the libraries obtained with this method was comparable to the quality of standard methods with minor constrains that are discussed in the manuscript. The dataset of the yeast segregants was used to determine recombination sites and chromosome copy number variations. With our large set of segregants we were able to study these processes in a quantitative way. Our recombination map correlated well with two independent datasets [17,18], suggesting a conservation of recombination distribution on a chromosome-wide scale among yeast strains. Furthermore, we detected chromosome-specific patterns of aneuploidy, which could be explained by structural variations between sister chromosomes and consequences of aneuploidy on fitness.

## Results and discussion

### DNA isolation

It is critical to disrupt the cell wall of *S. cerevisiae* before extracting genomic DNA (gDNA) from the cells. One can use either physical disruption (strong vortexing with glass beads [19]), or enzymes like zymolyase and lyticase [20]. Since strong vortexing in 96-well plates with phenol and glass beads can disrupt adhesiveness of the plate seals, this approach risks leakage and cross-contamination. Hence, we used enzymatic cell wall disruption for gDNA isolation. We isolated gDNA from up to 384 samples per day in 96-well format using a Biomek FX liquid handling robot. Combining cell pellets from 4 ml of overnight culture, the method yielded ~5.6 μg of DNA (average CV 2.6). This yield was slightly higher than from protocols that use glass beads [21,22] and a commercial column based method (“DNeasy 96 Blood & Tissue Kit”, Qiagen) in our hands, and was highly cost-effective (0.8 €/sample). For all tested protocols, the DNA was of high quality as determined by gel imaging and absorbance ratios (260/280 and 260/230 ratios 1.8 - 2.2). Furthermore, the isolated DNA contained enough mitochondrial DNA to genotype the mitochondrial genome in most of the segregants.

### DNA fragmentation

**Figure 2:**
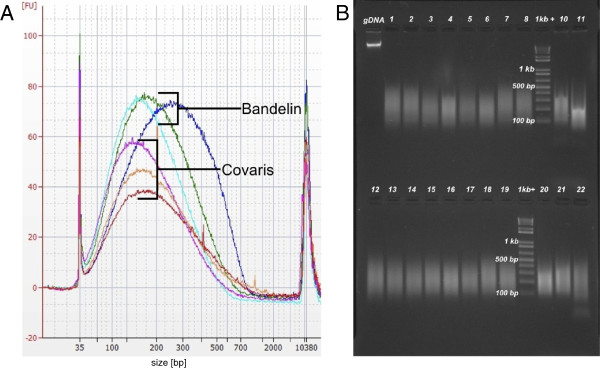
**Quality control of fragmented DNA. **(**A**) Bioanalyzer results from three DNA samples fragmented either in glass tubes with a Covaris DNA shearing device (Duty cycle 10%, Intensity 4.5, Cycles per burst 200, Time 120 s), or in PCR strips with a Bandelin sonicator (2 times 4 min). (**B**) 1.5% agarose gel loaded with 22 samples fragmented by Bandelin sonication. The size distribution is very narrow (major peak between 100–300 bp) and has an acceptable reproducibility.

**Figure 3:**
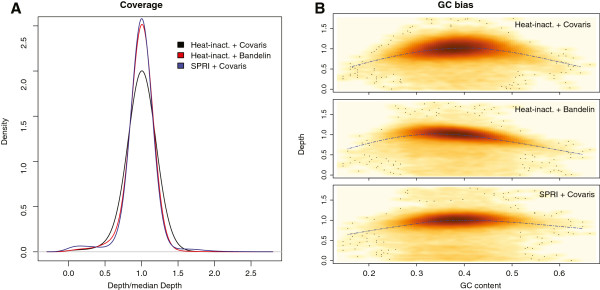
**Comparison of coverage homogeneity and GC bias between different techniques. **(**A**) The distribution of per-base depths was calculated (with only uniquely aligned reads) for our heat-inactivation protocol using either Covaris fragmentation (black) or Bandelin fragmentation (red), and is comparable to the standard library preparation, in which Covaris fragmentation was used in combination with SPRI cleanups (blue). (**B**) The GC bias is low for all compared techniques, as depicted on the right, with a slightly larger bias for the heat-inactivation protocols (using a mean depth of 200 bp bins, LOESS smooth with span = 0.3).

Fragmentation of DNA can be achieved by various methods, including transposon-based adapter insertion [23] and digestion with restriction enzymes [4]; however, physical fragmentation using AFA by Covaris is generally preferred because of its sharp, homogeneous, and random fragmentation [2]. Most of the samples in this study were fragmented using the Covaris E series, which allows automated processing of 96 samples; however, the initial capital and recurring expenses for the microTUBEs (~6 €/sample) makes this fragmentation method very expensive. A cheaper alternative is the use of PCR plates in combination with the Covaris machine instead of 96 microTUBES [6,7] or the use of a Bioruptor sonicator (Diagenode) [24] allowing for simultaneous sonication of 48 individual tubes. Here we present another method, using a sonicating water bath (Bandelin) in combination with two 8-PCR strips. We obtained DNA fragments that were similar in size range to Covaris sonication with sufficient reproducibility (Figure 2). The resultant sequencing reads were homogeneously distributed across the genome, and the GC bias was comparable to samples fragmented by Covaris sonication (Figure 3). Besides considerable cost reduction, this method has the advantage of working with a smaller volume of 25 μl (compared to 130 μl in Covaris tubes). Similar to Covaris sonication in 96-well plates [6,7], we occasionally observed samples that were not fragmented as efficiently as expected; therefore, analyzing the fragment sizes on an agarose gel prior to library preparation is recommended. We optimized the settings for 2 μg of DNA per sonication, which should be taken into consideration for studies where the DNA amount is a limiting factor.

### Library preparation

Most standard library preparation methods perform purification after blunt-ending, A-addition, and ligation steps to avoid carryover of enzymes. Many recent high-throughput protocols [4,6,11] have replaced column-based purifications and gel size selection steps with magnetic SPRI bead cleanups [25]. The reuse of the beads [5] and the use of a homemade bead mix [7] have also been applied to further reduce the cost of bead cleanups. Here we use heat inactivation, thereby circumventing purifications after blunt-ending, A-addition, and adapter ligation. This also reduces the risk of cross-contamination and sample loss during cleanups. The yield of the heat-inactivation protocol is comparable to the standard protocol (50- to 100-fold increase after PCR). <1% of read pairs have different barcodes on their forward and reverse sequences, indicating that the libraries obtained from this protocol have proper adapter ligation. The libraries are high-quality, with 87% mappability and 2.2% PCR duplicates, (detailed comparisons in Additional file 1: Table S2). In addition, the coverage of the *S. cerevisiae* genome yielded by our heat-inactivation protocol was highly uniform and comparable to libraries prepared with the standard Illumina protocol (Figure 3). A decrease in coverage was especially observed in regions with low GC content (<25%) when the heat-inactivation protocol was applied (examples are displayed in Additional file 2: Figure S1). This bias is slightly higher compared to the standard protocol (using SPRI cleanups), but was negligible for genotyping *S. cerevisiae*, as less than 0.5% of the 200 bp bins fall in this range. Genomes with a 30x coverage had 99% of the genome covered at > =1x and 97% at > =10x coverage. For the data shown here, we used 250 ng of fragmented DNA for the library preparation. We have also prepared libraries from starting amounts as low as 20 ng without a major loss in quality (see Additional file 1: Table S2 and Additional file 2: Figure S1). In principle, this would make the protocol compatible for RNA-Seq library preparations as well.

### Barcode bias

In this study, we used a set of 48 sequencing adapters containing 6 bp barcodes for ligation to the insert as reported by other groups [4,6-9,11]. After ligation, equimolar amounts of the barcoded libraries were pooled, size-selected, and amplified. The pooling of the samples before PCR resulted in moderately uneven barcode representation (Additional file 2: Figure S2), similar to previous reports [26,27]; this, however, did not adversely affect our genotyping quality. Seven barcodes that displayed extremely poor performance in the pool were excluded in our subsequent studies (Additional file 1: Table S3). We did not observe any particular pattern among the poorly performing barcodes, except that three of them had an “AA” before the T-overhang. No significant barcode bias was observed when the samples were amplified individually and pooled at equimolar concentration before size selection and sequencing (data not shown). For sample sets with limited DNA amounts, we would therefore recommend performing the PCRs individually and to pool equimolar amounts of samples prior to size selection.

### Recombination map

**Figure 4:**
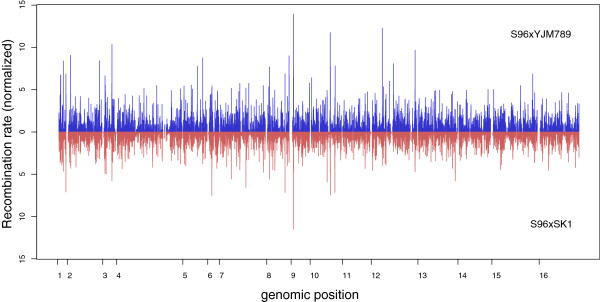
**Genome-wide recombination rate of *S. cerevisiae *segregants.** Recombination rate (normalized by the mean) of 184 segregants of an S96xYJM789 cross [17] are plotted in blue (top) and recombination rate from 720 segregants of an S96xSK1 cross (this study) are plotted in red (bottom) using a 2 kb window.

The sequenced yeast strains were haploid cells obtained by sporulation of a diploid hybrid of S96 (isogenic to S288c) and an SK1-derived strain (Mat_A, his3-Δ ura3-Δ can1-Δ flo8-Δ). After removing false positives, approximately 63,000 SNPs (~1 SNP every 190 bp) were used for genotyping. The average proportion of genotyped SNPs per segregant was 88.6%, and increased to 96.8% after imputation with Beagle [28]. With this dense marker set and 720 genotyped segregants (excluding 48 segregants with chromosomal aberrations and/or low read depth), we generated the highest-resolution recombination map to date (Figure 4, Additional file 1: Table S4). To compare our recombination map to a map previously generated from 50 tetrads in an S96xYJM789 cross [17], we estimated recombination rates directly from the genotypes of both datasets. We inferred a total of 50 recombination events per genome in our S96xSK1 segregants, which is significantly lower than the 63.2 recombination events inferred from the S96xYJM789 cross (P <2.2e-16). The total number of recombination events per genome estimated in our dataset is in closer agreement to the number reported by Martini et al. in an S96xSK1 cross [29] (43 recombination events per genome in a set of seven tetrads). The recombination rate in our S96xSK1 set is lower than the S96xYJM789 rate across all chromosomes and is therefore likely to be caused by differences in factors that globally affect meiotic recombination.

Recombination distributions between the S96xSK1 set and the S96xYJM789 set displayed a high correlation on a chromosome wide scale (0.944, P = 4.2e-08). To investigate possible differences on a local scale, we identified SNPs that are common between YJM789 and SK1 and then partitioned the S288c genome into non-overlapping bins (min 2 kb, max 3 kb) based on these SNPs. For this window size a lower correlation (0.616, P < 2.2e-16) was observed. A list of 20 regions with the largest differences in normalized recombination rates is provided in Additional file 1: Table S5.

Using the same partitioned bins as described above, we also compared the recombination rate with the genomic double-strand break (DSB) map generated by Pan et al. [18] (using immunoprecipitation of Spo11-bound oligos in meiotic SK1 cells). Similar to the comparison of our dataset with the S96xYJM789 dataset, we observed a good correlation on the chromosome-scale (0.726, P = 1.44e-03), but a lower one on the finer scale (0.375, P < 2.2e-16). These differences in hotspot intensities could be due to S96-specific hotspots or the possibility that not all DSBs lead to a detectable recombination event. Plotting the distance from the center of Spo11 oligo hotspots to the center of the S96xSK1 recombination events revealed a significant drop in recombination frequency in the vicinity (400-500 nucleotides) of the Spo11 hotspot (Additional file 2: Figure S3). This drop could be explained by the 5^′^ to 3^′^ resection of the resulting DNA ends, required for the repair of DSB by homologous recombination [30].

### Aneuploidy

**Figure 5:**
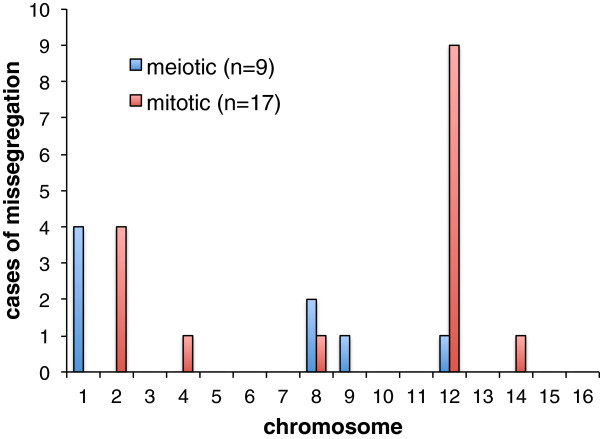
**Frequency of disomies across all chromosomes.** These disomies were detected in our set of 768 segregants and classified into missegregations during meiosis or mitosis depending on the respective allele frequencies (0.5 or 1) and copy number.

Using a window size of 10 kb, we generated coverage plots of all segregants (for examples see Additional file 2: Figure S4). In 3.6% of the segregants (n = 26), we observed an extra copy of a chromosome (including two with partial chromosome duplication). For nine of these segregants, the copy number of the affected chromosome was exactly two and had a 50% allele frequency (both SK1 and S96 alleles were present). These observations indicate a segregation error during the first meiotic division, in which one daughter cell received both sister chromosomes. Four of the nine disomies occurred in chromosome 1 (44%) (Figure 5). This chromosome might be particularly prone to missegregation because of its small size and substantial structural differences between the parental strains [31] (Additional file 2: Figure S5). In agreement with this explanation, a high degree of aneuploidy combined with low frequencies of genetic exchange has previously been observed in a cross between *S. cerevisiae* and *S. paradoxus*[32]. For the other 17 aneuploid strains, we observed homozygous calls and a copy number less than 2, suggesting that chromosomal duplications occurred after meiosis and only in a subpopulation of the particular segregant cells, which underwent 20–30 mitotic divisions before sequencing. Chromosome 12 had the highest rate of missegregation (53%). This might be due to the fact that it harbors the ribosomal gene cluster, which makes it the last chromosome to undergo segregation during mitosis [33,34]. Chromosome 12 disomy may also confer a growth advantage compared to other chromosomal duplications, which generally pose severe consequences or even lethality as reported for chromosome 6 [35,36].

## Conclusions

In this study, we present various optimization steps for whole DNA-Seq library preparation, considerably reducing the time and cost for library preparation compared to standard procedures. These include efficient high-throughput DNA isolation from yeast cells, a cost-effective alternative to standard Covaris fragmentation, and a library preparation that avoids most cleanup steps. The protocol was developed for the Illumina platform, but most of the steps are adaptable to other sequencing platforms with minor modifications. The quality of the DNA and final library was similar to that obtained by standard techniques. Although our heat inactivation step resulted in a slightly reduced coverage of regions with extreme GC content, this did not interfere with genotype calling. The genotype data was also used to map quantitative traits (Wilkening et al.*,* in revision), for which sample size and marker resolution are critical to maximize mapping resolution and statistical power. Furthermore, we created a map of meiotic recombination points in yeast with a yet unprecedented resolution as well as a catalog of chromosomal aberrations. Despite a high conservation of recombination at a chromosome-wide scale, our results indicate differences at the local scale. We also found an unexpectedly high degree of chromosomal aberrations in this genetic background. In conclusion, our method is a rapid, high-throughput approach for genotyping many small genomes or target-enriched DNA, and our results provide a unique basis for future and current studies of aneuploidy and recombination.

## Methods

### DNA isolation from yeast cells

A modified version of the PrepEase Genomic DNA Isolation Kit (Affymetrix, 78855 1 KT) based on enzymatic cell wall digestion was used for the DNA isolation from yeast cells. This protocol can easily be applied to blood, bacteria or homogenized tissue or plant material by substituting buffers according the manufacturer's instructions. All of the mixing steps were performed by pipetting using a Biomek FX pipetting robot (Beckman Coulter) in 96-well plates. Cell pellets from 4 deep-well plates, each containing 1 ml overnight culture, were combined for the DNA isolation. The “Spheroplast” and “Enzyme Solution” from the kit was replaced by Qiagen’s Y1 lysis buffer (1 M sorbitol, 100 mM EDTA, pH 8.0, 14 mM β-mercaptoethanol) freshly supplemented with 2.5 μl/ml of Zymolyase (Seikagaku Inc.) and 2.5 μl/ml RNase A (10 mg/ml, Qiagen). 200 μl of this buffer was added to each pellet, mixed and incubated at 37°C for 90 min with gentle shaking every 30 min. 200 μl of water was added to each well and the plate was centrifuged at 6000 x *g* for 4 min (for centrifuges with maximum 3,000 x *g*, centrifugation times can be tripled) and the supernatant was decanted. 120 μl Homogenization Buffer was added and mixed to resuspend the pellet completely. 100 μl of chloroform and 400 μl of Protein Precipitation Buffer were added to the lysate and mixed. Plates were centrifuged at 6,000 × g for 15 min. 450 μl of the upper aqueous phase was transferred with the robot (pipetting height was optimized in advance) to a 1 ml deep-well plate containing 340 μl of isopropanol per well. The solution was mixed, left for 15 min at room temperature, and centrifuged at 6,000 × *g* for 15 min. After decanting the supernatant, 1 ml of cold 70% ethanol was added to the pellet, mixed and centrifuged at 6,000 × *g* for 10 min. The supernatant was decanted, and the tube was placed upside down on a paper towel and dried for 5 min at 37°C. The DNA pellet was resuspended in 300 μl of DNA Resuspension Buffer or Elution Buffer (EB, 10 mM Tris HCl) by shaking plates for 30 min at 37°C and later by mixing. A detailed Biomek protocol including .bmf files is provided in Additional file 3.

### DNA fragmentation

1–10 μg of genomic DNA from each of the 768 segregants was sheared using a Covaris E series sonicator in 130 μl to obtain a fragment size with a major peak of ~250 bp (Duty cycle 10%, Intensity 4.5, Cycles per burst 200, 120 sec). Samples from Covaris sonication were transferred to 96-well PCR plates, dried in a Speedvac, and resuspended in 30 μl of EB. Alternatively, we tested sonication in PCR-strips using a Sonorex RK 102 sonicating water bath (Bandelin). For this, two 8-strips held on a support plate were fixed to a cycling pin that rotates during sonication (Additional file 4: Video S1). After 4 min sonication at 4°C, samples were spun down and sonicated for another 4 min. A uniform size distribution was obtained by keeping the volume and DNA amount constant (2 μg in 25 μl). All samples were run on a 1.5% agarose gel to verify the fragment size.

### End repair, dA-tailing, and ligation using heat-inactivation

Instead of the standard column or bead-based cleanup steps, we heat-inactivated the enzymes used for end repair, dA-tailing, and ligation, then added the respective enzyme (+ buffer). For this, 250 ng of fragmented gDNA were used for the library preparation in 96-well PCR plates in a volume of 17 μl. End repair for the fragments was performed by adding 3 μl of End repair master mix composed of 2 μl of End repair buffer and 1 μl End repair enzyme (NEBNext End Repair Module, NEB #E6050L) using a 8-channel pipette. The contents were mixed by vortexing, shortly spun down, and incubated in a thermocycler at 20°C for 45 min. The enzymes were then heat-inactivated at 75°C for 15 min. The contents of the plate were quickly spun down, and 2 μl of A tailing master mix containing 1 μl of Klenow Fragment (3′→5′ exo–) (NEB #M0212L), 0.5 μl of nuclease free water, and 0.5 μl of 100 mM dATP (NEB #N0440S) were added to the 20 μl reaction. The contents were mixed by vortexing, spun down, and incubated in a thermocycler at 37°C for 45 minutes. The enzymes were then heat-inactivated at 75°C for 15 minutes. 5 μl of ligation master mix containing 3 μl 10X T4 DNA ligase buffer and 2 μl T4 DNA ligase (NEB #M0202L) were added to the reaction followed by 3 μl of 7 μM multiplex barcode adapters (aliquoted into 8-strip PCR tubes or 96-well plates for convenient pipetting, see Additional file 1: Table S2 for sequences). The concentration of adapters was optimized to reduce the formation of adapter dimers. The reaction contents were mixed well, spun down, and incubated on a thermocycler at 16°C for 1 h followed by heat inactivation at 75°C for 15 min.

### Pooling and size selection

After barcode ligation and heat inactivation, 48 samples were pooled together by combining 5 μl of each sample in a 1.5 ml reaction tube. The samples were cleaned up and concentrated to 40 μl using 1x Ampure XP cleanup. This pooling step reduces the sample size from 96 to two for the subsequent size selection and PCR. For size selection, 25 μl (roughly 1.25 μg) of ligated DNA was loaded on a 2% E-Gel SizeSelect (Invitrogen) and DNA fragments were collected at 350 bp and 400 bp. The DNA concentrations were then determined by Qubit HS DNA reagent (Invitrogen).

### PCR enrichment

In a 50 μl reaction, 5–10 ng of the pooled libraries were amplified. We have observed that performing PCR with an excess of template DNA (>20 ng) significantly reduces the efficiency of the PCR. The PCR was performed on a thermocycler (MJ Research tetrad) containing 1x Phusion Master Mix with HF Buffer (Thermo Scientific) and 0.2 μM Illumina PE 1.0 and 2.0 primers. The low primer concentration reduced the formation of primer dimers often observed at standard primer concentrations (1.25 μM). PCR conditions were 98°C for 45 s, 10x [98°C for 15 s, 65°C for 30 s, 72°C for 30 s], 72°C for 5 min, 4°C hold.

### DNA purification with SPRI beads

The amplified DNA was purified in 0.2 ml PCR strips by mixing the DNA with 1x volume of Agencourt AMPure XP beads (Beckman Coulter) and select the magnetic beads with a homemade magnetic stand (Additional file 2: Figure S6). This stand consists of neodymium magnets (Webcraft GmbH, Gottmadingen, Germany) mounted on trimmed 96-well plates and can be used in combination with an 8-channel pipet. For a high-throughput SPRI clean-up we further provide a detailed protocol of the pipetting steps for 96-well plates and Biomek robot in Additional file 3. DNA concentrations were quantified for the subsequent library preparation (see DNA quantification and quality control).

### DNA quantification and quality control

The quality of individual samples of isolated DNA was determined by a photospectrometric measurement using a NanoDrop 1000 (Thermo Fisher). For quantification of genomic DNA and pre-PCR libraries in 96-well plates, we used Quant-iT PicoGreen dsDNA Reagent (Invitrogen) in optical plates (Greiner). The fluorescence was measured at 485 nm excitation and 535 nm emission in a Genios microplate reader (Tecan) according to the manufacturer’s instructions. The pooled libraries were quantified before and after PCR with a Qubit spectrofluorometer (Invitrogen) according to the manufacturer’s instructions. All pre- and post-PCR libraries were run on a High Sensitivity Bioanalyzer chip (Agilent) to determine the size distribution. After a 10-cycle PCR, we typically observed a 10-fold increase in DNA amount and a 24–30 bp increase in library size due to adapter elongation. Depending on the amplification efficiency, either the low (350 bp) or the high molecular weight (400 bp) library was selected for sequencing. The samples were then diluted to 10 nM and clustered on the Illumina cBot clustering station for paired-end sequencing on an Illumina HiSeq 2000.

### Design of 48 multiplexing barcodes

We designed a set of 48 adapters, each with a different hexamer sequence just before the T-overhang, similar to Lefrancois et al [9]. We selected 64 of 96 Illumina barcodes, which had at least a 3 bp difference compared to any of the other 63 barcodes. From this set, 48 barcodes that had an equilibrated base composition at the first two bases (for better cluster calling) were manually chosen. Following quality control analysis, we replaced the seven poorest performing barcodes with new ones (Additional file 1: Table S2).

### Genotyping

To demultiplex, we extracted the first six bases of each read and compared it to all possible barcodes. The perfect match or best hit to one barcode with the least number of mismatches was assigned to the read. For genotyping, reads from the segregants along with both SK1 and S96 (a haploid strain isogenic to S288c) parental strains were aligned to the S288c reference genome (build R63) using Novoalign (v2.07.06; http://www.novocraft.com/), allowing for unique alignments. Thereafter GATK was used for realignment and recalibration of the bam files [37], and subsequent SNP calling was performed using SAMtools [38]. The vcf file produced by SAMtools contains a list of variant positions and the individual genotype calls across all samples at each variant position. The formula that SAMtools applies for calling the genotype is dependent on allele frequency, which is not directly applicable to our study, because the allele frequency at true SNP positions is expected to be 0.5 in crosses generated from 2 parents. Instead, we used the genotype likelihood (PL stats generated by GATK) to infer the genotype. SNP positions, which correspond to a homozygous reference call in the S96 parent and a homozygous variant in the SK1 parent, are chosen first. From this set of SNPs, we excluded calls whose allele frequency is not between 0.3-0.7. These SNP calls are unreliable and often not in linkage with their surrounding SNPs, and could either be SNPs within regions that are repetitive in one but not in the other parent, or result from misaligned reads.

### GC bias and coverage plots

We calculated the genome-wide, per-base coverage of the S288c genome using SAMtools. Positions where all samples had at least 1 read were considered. The density was plotted with a bandwidth of 0.1. For plotting GC bias, the genome was divided into non-overlapping 200 bp bins, and the depth was estimated by the mean values of per-base depth in these bins. Bins with less than 50% covered by at least 1 read were excluded. All analyses were run in the software R (v. 2.12.0; http://cran.r-project.org). For analyzing chromosomal abnormalities, an identical method for binning and GC correction was applied, except that a 10 kb bin size was used. GC bias correction was applied using a LOESS method, as described previously [39].

### Recombination map analysis

For both genotype datasets (S96xSK1 and S96xYJM789) the rqtl package (with the function, est.map (maxit = 1000,error.prob = 0.01) was used to construct the genetic map for both crosses. After obtaining the genetic map, the genotypes were filtered for errors and crossovers counted for each segregant, using functions in rqtl (cleanGeno(maxdist = 2.5, maxmark = 2) followed by countXO). For 2-3 kb bins (partitioned by common SNPs), the recombination rate was calculated as genetic distance between 2 SNPs/physical distance between 2 SNPs. For identifying regions with difference in recombination rates, we normalized the rate in both, S96xSK1 and S96xYJM789 by setting the mean of each set to 1. Raw sequences for Spo11 oligo maps were download from SRA (GSE26452) and aligned to the S288c genome build R63 using bowtie2 allowing for only unique alignments.

## Abbreviations

NGS: Next-generation sequencing; DNA-Seq: NGS of genomic DNA; SPRI: Solid phase reversible immobilization; AFA: Adaptive Focused Acoustics; gDNA: Genomic DNA; GATK: Genome Analysis Toolkit; vcf: Variant call format; DSB: Double strand break; CV: Coefficient of variation.

## Competing interests

The authors declare that they have no competing interests.

## Authors’ contributions

SW and MMT developed and carried out the experimental work; GL performed the all sequence alignment and did most of the statistical analyses; ESF, WW, and JG contributed to the statistical analysis; DWL and AC developed the heat-inactivation steps; SW, MMT, and LMS conceived the study and wrote the manuscript. All authors reviewed the draft, contributed comments, and approved the final manuscript.

## Supplementary Material

Additional file 1: Tables S1 Summary of publications on DNA-Seq improvements, **Tables S2.** Quality measures for different library preparations, **Tables S3.** Oligonucleotide sequences, **Tables S4.** Recombination sites in SK1xS96 segregants, **Tables S5.** List of 20 regions with biggest differences between recombination frequency between S96xYJM789 and SK1xS96 set. Click here for file

Additional file 2: Figures S1 IGV example for regions with extreme GC content, **Figures S2.** Barcode amplification bias, **Figures S3.** Recombination frequency around Spo11 oligo hotspots, **Figures S4.** Detection and classification of missegregation, **Figures S5.** Correlation between meiotic disomy and structural variations, **Figures S6.** Photo of self-made magnetic stand. Click here for file

Additional file 3 bmf files (zipped files including + protocol). Click here for file

Additional file 4: Video S1 Showing fragmentation by Bandelin sonication. Click here for file
