# Supplementary material for: Genotyping 1000 yeast strains by next-generation sequencing
Source: BMC Genomics. 2013 Feb 9;14:90. doi: 10.1186/1471-2164-14-90 (PMC3575377; doi:10.1186/1471-2164-14-90)
Supplement: Additional file 2: Figures S1 — IGV example for regions with extreme GC content, Figures S2. Barcode amplification bias, Figures S3. Recombination frequency around Spo11 oligo hotspots, Figures S4. Detection and classification of missegregation, Figures S5. Correlation between meiotic disomy and structural variations, Figures S6. Photo of self-made magnetic stand. [file 1471-2164-14-90-S2.doc]

Supplementary Figures

**Supplementary Figure 1**: Coverage of example regions with extreme GC content (marked in red) are displayed in IGV for a library prepared from (a) 20ng DNA, (b) by heat inactivation (HI) + Bandelin sonicator, (c) by HI + Covaris machine, (d) standard protocol. Little loss of coverage was seen at the high GC region (upper panel with 57% GC at chr01:187,559-195,646). A HI specific loss of coverage could be seen in a region with low GC content (lower panel with 21% GC at chr07:89,500-89,892).


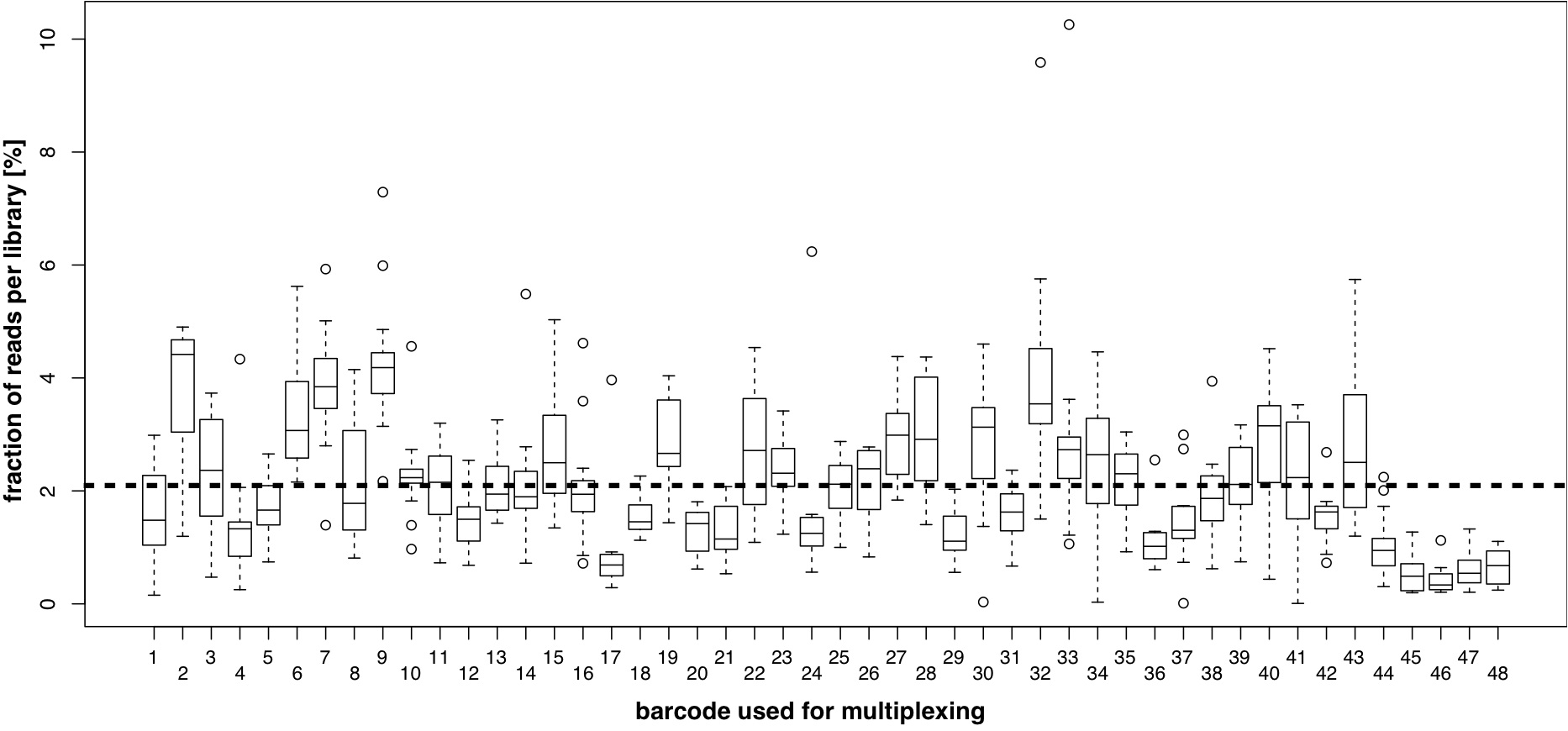


**Supplementary Figure 2**: Box plot of fraction of reads for each of the 48 multiplex adapters used for library preparation across 16 HiSeq lanes. The dashed line indicates the percentage of reads expected for a perfect barcode distribution (2.08 %).


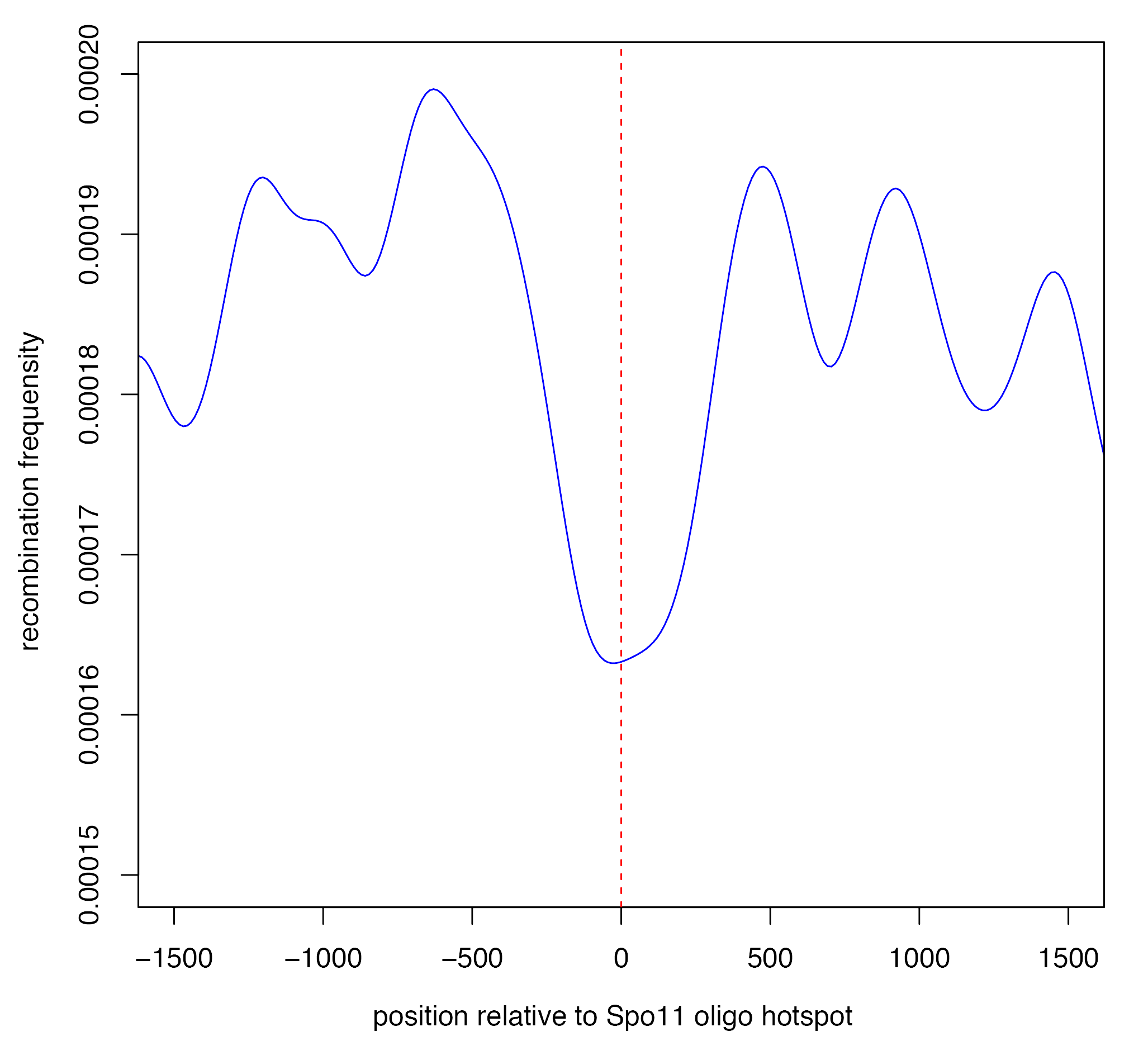


**Supplementary Figure 3**: Plot of recombination frequency around Spo11 oligo hotspots (N = 65,578, bandwidth of 100bp) as reported by Pan et al.


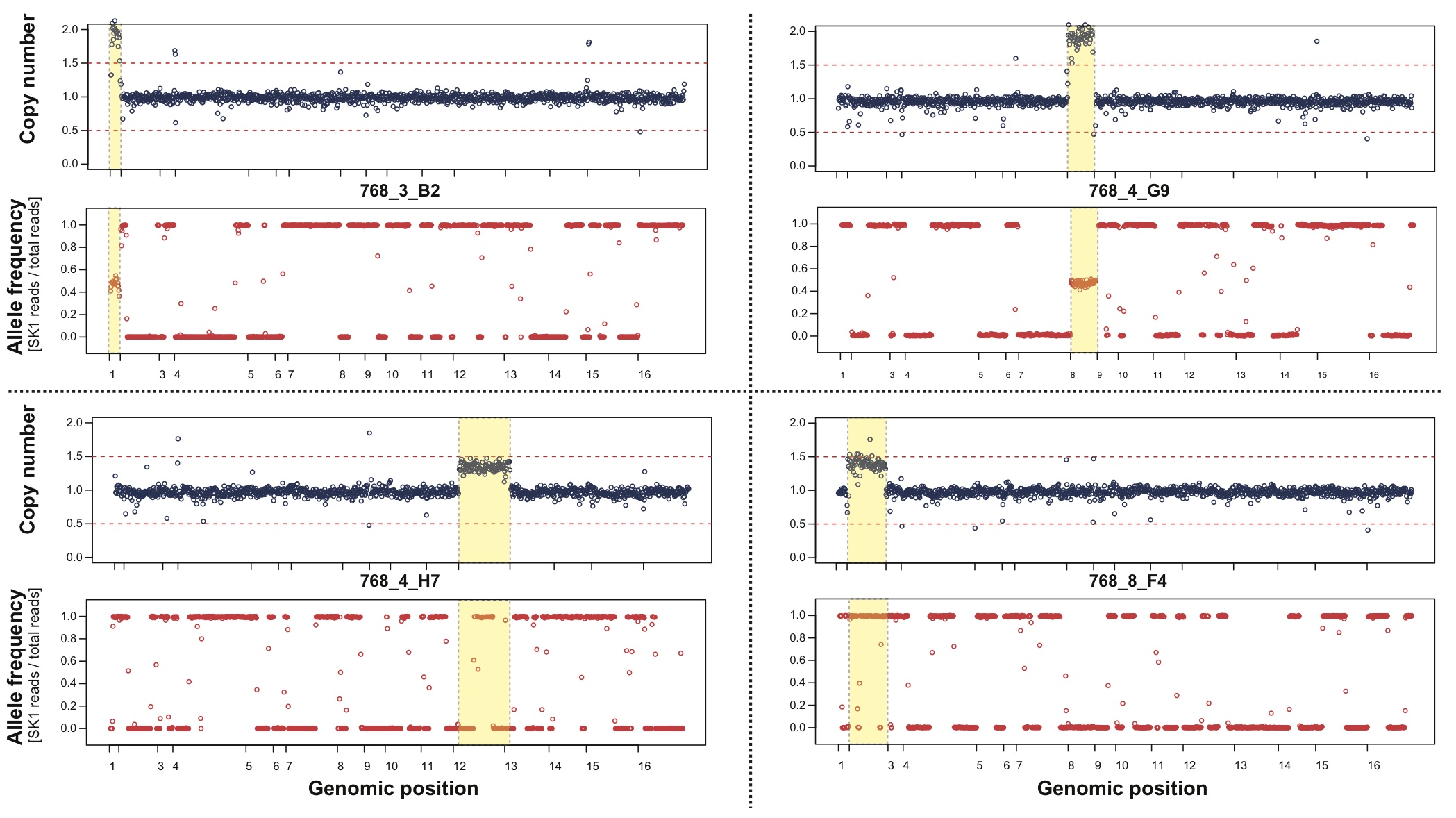


**Supplementary Figure 4**: Examples of aneuploidy detection in four different segregants. Genome-wide coverage is depicted in blue and SK1 allele frequency in red. The upper two samples show evidence for chromosomal missegregation during meiosis leading to disomy: a two-fold coverage and allele frequency of 0.5. The lower two samples show evidence for chromosome duplication during mitotic division: a partial copy number increase and homozygous genotype (allele frequency of 0 or 1).

**Supplementary Figure 5**: Plot showing cases of disomy vs. percentage of regions deleted in SK1 relative to S288c according to Schacherer et al. 2009 . Chromosomes with bigger structural variation tend to have more cases of disomy.


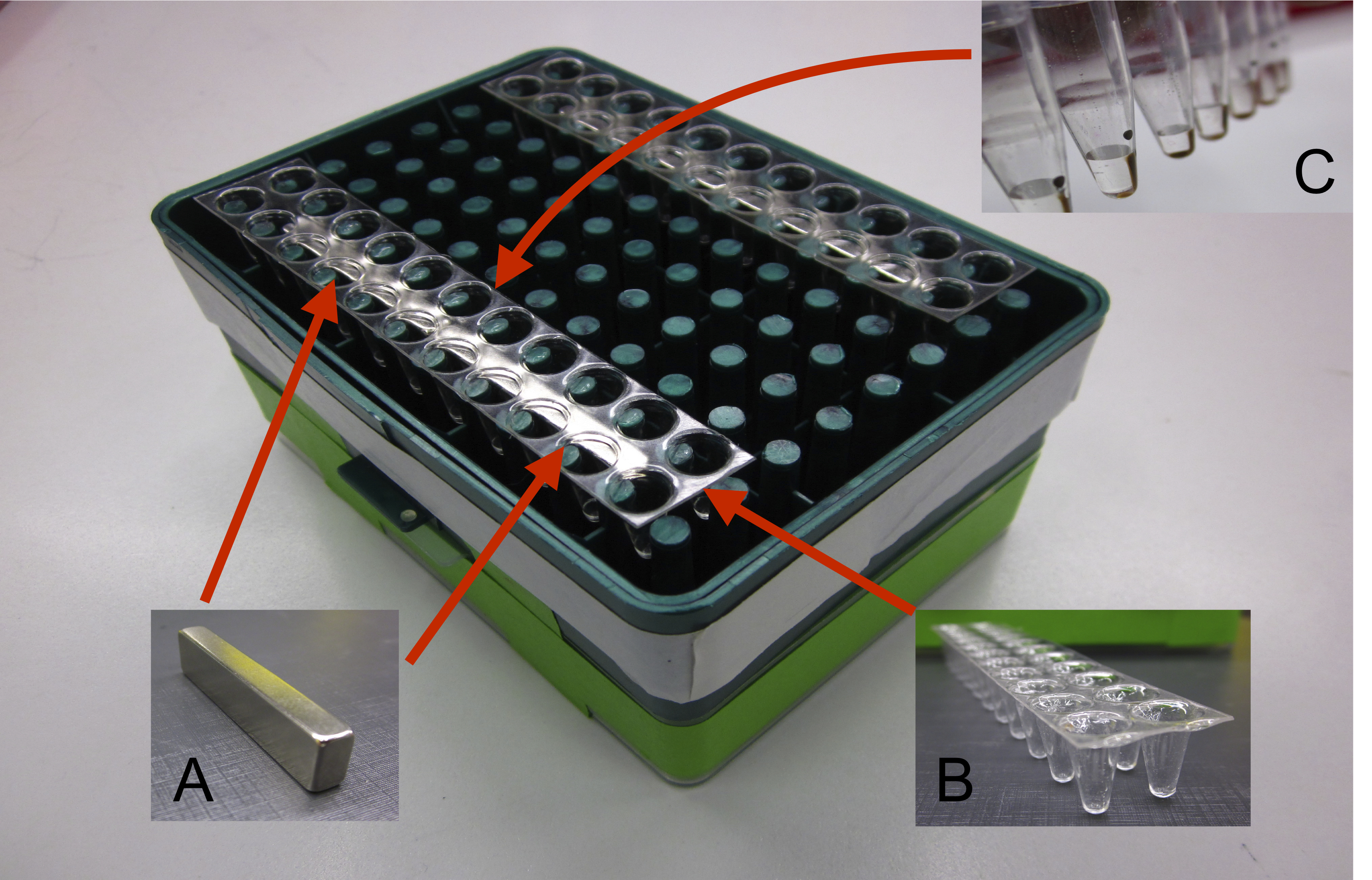


**Supplementary Figure 6:** Home-made magnetic stand for SPRI bead purification. An upside down tip box was used as the base to hold a bar magnet (A) and a trimmed 96 well plate (B), serving as a rack for PCR strips containing magnetic beads and sample (C) (adapted from Pelechano *et al.* ).

**Supplementary References**

1. Robinson JT, Thorvaldsdottir H, Winckler W, Guttman M, Lander ES, Getz G, Mesirov JP: **Integrative genomics viewer.** *Nat Biotechnol* 2011, **29:**24-26.

2. Pan J, Sasaki M, Kniewel R, Murakami H, Blitzblau HG, Tischfield SE, Zhu X, Neale MJ, Jasin M, Socci ND, et al: **A hierarchical combination of factors shapes the genome-wide topography of yeast meiotic recombination initiation.** *Cell* 2011, **144:**719-731.

3. Schacherer J, Shapiro JA, Ruderfer DM, Kruglyak L: **Comprehensive polymorphism survey elucidates population structure of Saccharomyces cerevisiae.** *Nature* 2009, **458:**342-345.

4. Pelechano V, Wilkening S, Järvelin AI, Tekkedil MM, Steinmetz LM: **Genome-wide polyadenylation site mapping.** In *Nucleosomes, Histones & Chromatin, Part B.* *Volume* 513. Edited by Carl Wu CDA; 2012: accepted
