# Supplementary material for: Genotyping 1000 yeast strains by next-generation sequencing
Source: BMC Genomics. 2013 Feb 9;14:90. doi: 10.1186/1471-2164-14-90 (PMC3575377; doi:10.1186/1471-2164-14-90)
Supplement: Additional file 1: Tables S1 — Summary of publications on DNA-Seq improvements, Tables S2. Quality measures for different library preparations, Tables S3. Oligonucleotide sequences, Tables S4. Recombination sites in SK1xS96 segregants, Tables S5. List of 20 regions with biggest differences between recombination frequency between S96xYJM789 and SK1xS96 set. [file 1471-2164-14-90-S1.zip › supplementary_Tables/Supplementary Table 2.doc]

| **Sample** | **PCR duplicates ^1)^** | | **library size ^1)^** | | **mapped to S288c ^2)^** | | **chr. Chimera ^2)^** | |
| --- | --- | --- | --- | --- | --- | --- | --- | --- |
|  | mean | CV | mean | CV | mean | CV | mean | CV |
| HI + Bandelin | 0.022 | 0.26 | 118,856,938 | 0.59 | 0.88 | 0.08 | 0.00056 | 0.19 |
| HI + Covaris | 0.029 | 0.59 | 101,875,048 | 0.80 | 0.85 | 0.01 | 0.00030 | 0.91 |
| SPRI + Covaris | 0.049 | 0.09 | 119,739,670 | 0.57 | 0.87 | 0.02 | 0.00079 | 0.39 |
| 20ng HI + Bandelin | 0.087 | not cal. ^3)^ | 680,774 | not cal. ^3)^ | 0.76 | not cal. ^3)^ | 0.00097 | not cal. ^3)^ |

1) calculated using picard tools
2) calculated using samtools flagstat
3) since we only have two samples we did not calculate the CV *(*coefficient of variation)
