# Supplementary material for: Genotyping 1000 yeast strains by next-generation sequencing
Source: BMC Genomics. 2013 Feb 9;14:90. doi: 10.1186/1471-2164-14-90 (PMC3575377; doi:10.1186/1471-2164-14-90)
Supplement: Additional file 1: Tables S1 — Summary of publications on DNA-Seq improvements, Tables S2. Quality measures for different library preparations, Tables S3. Oligonucleotide sequences, Tables S4. Recombination sites in SK1xS96 segregants, Tables S5. List of 20 regions with biggest differences between recombination frequency between S96xYJM789 and SK1xS96 set. [file 1471-2164-14-90-S1.zip › supplementary_Tables/Supplementary Table 3.doc]

**Supplementary Table1:** Oligonucleotide sequences

Sequences of the oligonucleotides used for this protocol are listed below and sorted in a base equilibrated order for the use of less then 48 barcodes (i.e. for 8 samples, use barcode 01-08). The multiplex barcode sequences are underlined. All oligos were purchased in a HPLC purified format from Sigma-Aldrich, Germany.

| **no.** | **barcode** | **bad_oligo** | **upper_oligo** | **lower_oligo** |
| --- | --- | --- | --- | --- |
| 01 | CTAAGG |  | ACACTCTTTCCCTACACGACGCTCTTCCGATCTCTAAGG*T | [Phos]CCTTAGAGATCGGAAGAGCGGTTCAGCAGGAATGCCGAG |
| 02 | ATTGGC | CCAGCA | ACACTCTTTCCCTACACGACGCTCTTCCGATCTATTGGC*T | [Phos]GCCAATAGATCGGAAGAGCGGTTCAGCAGGAATGCCGAG |
| 03 | CATTCA |  | ACACTCTTTCCCTACACGACGCTCTTCCGATCTCATTCA*T | [Phos]TGAATGAGATCGGAAGAGCGGTTCAGCAGGAATGCCGAG |
| 04 | TGTGCC |  | ACACTCTTTCCCTACACGACGCTCTTCCGATCTTGTGCC*T | [Phos]GGCACAAGATCGGAAGAGCGGTTCAGCAGGAATGCCGAG |
| 05 | CACTGT |  | ACACTCTTTCCCTACACGACGCTCTTCCGATCTCACTGT*T | [Phos]ACAGTGAGATCGGAAGAGCGGTTCAGCAGGAATGCCGAG |
| 06 | TGGAGC |  | ACACTCTTTCCCTACACGACGCTCTTCCGATCTTGGAGC*T | [Phos]GCTCCAAGATCGGAAGAGCGGTTCAGCAGGAATGCCGAG |
| 07 | GCTACC |  | ACACTCTTTCCCTACACGACGCTCTTCCGATCTGCTACC*T | [Phos]GGTAGCAGATCGGAAGAGCGGTTCAGCAGGAATGCCGAG |
| 08 | CGTACG |  | ACACTCTTTCCCTACACGACGCTCTTCCGATCTCGTACG*T | [Phos]CGTACGAGATCGGAAGAGCGGTTCAGCAGGAATGCCGAG |
| 09 | ATTATA |  | ACACTCTTTCCCTACACGACGCTCTTCCGATCTATTATA*T | [Phos]TATAATAGATCGGAAGAGCGGTTCAGCAGGAATGCCGAG |
| 10 | TACAAG |  | ACACTCTTTCCCTACACGACGCTCTTCCGATCTTACAAG*T | [Phos]CTTGTAAGATCGGAAGAGCGGTTCAGCAGGAATGCCGAG |
| 11 | GTATAG |  | ACACTCTTTCCCTACACGACGCTCTTCCGATCTGTATAG*T | [Phos]CTATACAGATCGGAAGAGCGGTTCAGCAGGAATGCCGAG |
| 12 | GGAACT |  | ACACTCTTTCCCTACACGACGCTCTTCCGATCTGGAACT*T | [Phos]AGTTCCAGATCGGAAGAGCGGTTCAGCAGGAATGCCGAG |
| 13 | CCTTGC |  | ACACTCTTTCCCTACACGACGCTCTTCCGATCTCCTTGC*T | [Phos]GCAAGGAGATCGGAAGAGCGGTTCAGCAGGAATGCCGAG |
| 14 | CGCCTG |  | ACACTCTTTCCCTACACGACGCTCTTCCGATCTCGCCTG*T | [Phos]CAGGCGAGATCGGAAGAGCGGTTCAGCAGGAATGCCGAG |
| 15 | ATCGTG |  | ACACTCTTTCCCTACACGACGCTCTTCCGATCTATCGTG*T | [Phos]CACGATAGATCGGAAGAGCGGTTCAGCAGGAATGCCGAG |
| 16 | GCTCAT |  | ACACTCTTTCCCTACACGACGCTCTTCCGATCTGCTCAT*T | [Phos]ATGAGCAGATCGGAAGAGCGGTTCAGCAGGAATGCCGAG |
| 17 | CAGGCC |  | ACACTCTTTCCCTACACGACGCTCTTCCGATCTCAGGCC*T | [Phos]GGCCTGAGATCGGAAGAGCGGTTCAGCAGGAATGCCGAG |
| 18 | CAAGTT |  | ACACTCTTTCCCTACACGACGCTCTTCCGATCTCAAGTT*T | [Phos]AACTTGAGATCGGAAGAGCGGTTCAGCAGGAATGCCGAG |
| 19 | AGTCTT |  | ACACTCTTTCCCTACACGACGCTCTTCCGATCTAGTCTT*T | [Phos]AAGACTAGATCGGAAGAGCGGTTCAGCAGGAATGCCGAG |
| 20 | AAGCTA | TTCGAA | ACACTCTTTCCCTACACGACGCTCTTCCGATCTAAGCTA*T | [Phos]TAGCTTAGATCGGAAGAGCGGTTCAGCAGGAATGCCGAG |
| 21 | AGCTAG |  | ACACTCTTTCCCTACACGACGCTCTTCCGATCTAGCTAG*T | [Phos]CTAGCTAGATCGGAAGAGCGGTTCAGCAGGAATGCCGAG |
| 22 | GTAGCC | GCCTAA | ACACTCTTTCCCTACACGACGCTCTTCCGATCTGTAGCC*T | [Phos]GGCTACAGATCGGAAGAGCGGTTCAGCAGGAATGCCGAG |
| 23 | CGAAAC |  | ACACTCTTTCCCTACACGACGCTCTTCCGATCTCGAAAC*T | [Phos]GTTTCGAGATCGGAAGAGCGGTTCAGCAGGAATGCCGAG |
| 24 | CTCTAC |  | ACACTCTTTCCCTACACGACGCTCTTCCGATCTCTCTAC*T | [Phos]GTAGAGAGATCGGAAGAGCGGTTCAGCAGGAATGCCGAG |
| 25 | CCGTAT |  | ACACTCTTTCCCTACACGACGCTCTTCCGATCTCCGTAT*T | [Phos]ATACGGAGATCGGAAGAGCGGTTCAGCAGGAATGCCGAG |
| 26 | TATATC |  | ACACTCTTTCCCTACACGACGCTCTTCCGATCTTATATC*T | [Phos]GATATAAGATCGGAAGAGCGGTTCAGCAGGAATGCCGAG |
| 27 | TCCGTC |  | ACACTCTTTCCCTACACGACGCTCTTCCGATCTTCCGTC*T | [Phos]GACGGAAGATCGGAAGAGCGGTTCAGCAGGAATGCCGAG |
| 28 | TCTGAG |  | ACACTCTTTCCCTACACGACGCTCTTCCGATCTTCTGAG*T | [Phos]CTCAGAAGATCGGAAGAGCGGTTCAGCAGGAATGCCGAG |
| 29 | ACATCG | ATGGCA | ACACTCTTTCCCTACACGACGCTCTTCCGATCTACATCG*T | [Phos]CGATGTAGATCGGAAGAGCGGTTCAGCAGGAATGCCGAG |
| 30 | AAGTGC |  | ACACTCTTTCCCTACACGACGCTCTTCCGATCTAAGTGC*T | [Phos]GCACTTAGATCGGAAGAGCGGTTCAGCAGGAATGCCGAG |
| 31 | CTGATC |  | ACACTCTTTCCCTACACGACGCTCTTCCGATCTCTGATC*T | [Phos]GATCAGAGATCGGAAGAGCGGTTCAGCAGGAATGCCGAG |
| 32 | TGAGTG |  | ACACTCTTTCCCTACACGACGCTCTTCCGATCTTGAGTG*T | [Phos]CACTCAAGATCGGAAGAGCGGTTCAGCAGGAATGCCGAG |
| 33 | TGGTCA | GGAGAA | ACACTCTTTCCCTACACGACGCTCTTCCGATCTTGGTCA*T | [Phos]TGACCAAGATCGGAAGAGCGGTTCAGCAGGAATGCCGAG |
| 34 | GCGTGG |  | ACACTCTTTCCCTACACGACGCTCTTCCGATCTGCGTGG*T | [Phos]CCACGCAGATCGGAAGAGCGGTTCAGCAGGAATGCCGAG |
| 35 | CGTGAT | TTGCGG | ACACTCTTTCCCTACACGACGCTCTTCCGATCTCGTGAT*T | [Phos]ATCACG AGATCGGAAGAGCGGTTCAGCAGGAATGCCGAG |
| 36 | GAGAGT |  | ACACTCTTTCCCTACACGACGCTCTTCCGATCTGAGAGT*T | [Phos]ACTCTCAGATCGGAAGAGCGGTTCAGCAGGAATGCCGAG |
| 37 | GATCTG | GGCCAC | ACACTCTTTCCCTACACGACGCTCTTCCGATCTGATCTG*T | [Phos]CAGATCAGATCGGAAGAGCGGTTCAGCAGGAATGCCGAG |
| 38 | ATTCCG |  | ACACTCTTTCCCTACACGACGCTCTTCCGATCTATTCCG*T | [Phos]CGGAATAGATCGGAAGAGCGGTTCAGCAGGAATGCCGAG |
| 39 | AGCGCT |  | ACACTCTTTCCCTACACGACGCTCTTCCGATCTAGCGCT*T | [Phos]AGCGCTAGATCGGAAGAGCGGTTCAGCAGGAATGCCGAG |
| 40 | GCTGTA |  | ACACTCTTTCCCTACACGACGCTCTTCCGATCTGCTGTA*T | [Phos]TACAGCAGATCGGAAGAGCGGTTCAGCAGGAATGCCGAG |
| 41 | TCGCTT |  | ACACTCTTTCCCTACACGACGCTCTTCCGATCTTCGCTT*T | [Phos]AAGCGAAGATCGGAAGAGCGGTTCAGCAGGAATGCCGAG |
| 42 | TGCCGA |  | ACACTCTTTCCCTACACGACGCTCTTCCGATCTTGCCGA*T | [Phos]TCGGCAAGATCGGAAGAGCGGTTCAGCAGGAATGCCGAG |
| 43 | GAATGA |  | ACACTCTTTCCCTACACGACGCTCTTCCGATCTGAATGA*T | [Phos]TCATTCAGATCGGAAGAGCGGTTCAGCAGGAATGCCGAG |
| 44 | CCACTC |  | ACACTCTTTCCCTACACGACGCTCTTCCGATCTCCACTC*T | [Phos]GAGTGGAGATCGGAAGAGCGGTTCAGCAGGAATGCCGAG |
| 45 | GATGCT |  | ACACTCTTTCCCTACACGACGCTCTTCCGATCTGATGCT*T | [Phos]AGCATCAGATCGGAAGAGCGGTTCAGCAGGAATGCCGAG |
| 46 | TGACAT |  | ACACTCTTTCCCTACACGACGCTCTTCCGATCTTGACAT*T | [Phos]ATGTCAAGATCGGAAGAGCGGTTCAGCAGGAATGCCGAG |
| 47 | ATAGAT |  | ACACTCTTTCCCTACACGACGCTCTTCCGATCTATAGAT*T | [Phos]ATCTATAGATCGGAAGAGCGGTTCAGCAGGAATGCCGAG |
| 48 | TCGGGA |  | ACACTCTTTCCCTACACGACGCTCTTCCGATCTTCGGGA*T | [Phos]TCCCGAAGATCGGAAGAGCGGTTCAGCAGGAATGCCGAG |

Modifications are marked as followed: * Phosphorothioate linkage, [Phos] 5’Phosphorilation.

**PCR primers**

| PE 1.0 Illumina^1^ | AATGATACGGCGACCACCGAGATCTACACTCTTTCCCTACACGACGCTCTTCCGATCT |
| --- | --- |
| PE 2.0 Illumina^1^ | CAAGCAGAAGACGGCATACGAGATCGGTCTCGGCATTCCTGCTGAACCGCTCTTCCGATCT |

^1^ Oligonucleotide sequences are property of Illumina, Inc.
