# Supplementary material for: Genotyping 1000 yeast strains by next-generation sequencing
Source: BMC Genomics. 2013 Feb 9;14:90. doi: 10.1186/1471-2164-14-90 (PMC3575377; doi:10.1186/1471-2164-14-90)
Supplement: Additional file 1: Tables S1 — Summary of publications on DNA-Seq improvements, Tables S2. Quality measures for different library preparations, Tables S3. Oligonucleotide sequences, Tables S4. Recombination sites in SK1xS96 segregants, Tables S5. List of 20 regions with biggest differences between recombination frequency between S96xYJM789 and SK1xS96 set. [file 1471-2164-14-90-S1.zip › supplementary_Tables/Supplementary Table 1.doc]

**Supplementary Table 1:** Summary of studies with innovations to library preparation of high-throughput DNA-Seq.

| **Study** | **Journal** | **Year** | **DNA isolation or capture** | **Fragmentation** | **Adapter ligation** | **Barcoding** | **Size selection** | **Pooling** | **Platform** |
| --- | --- | --- | --- | --- | --- | --- | --- | --- | --- |
| Quail et al. | *Nature Methods* | 2008 | various | Covaris 24-well format | standard | no barcoding | gel extraction (no heating) | not applied (1 sample/lane) | GAII |
| Lefrancois et al. | *BMC Genomics* | 2009 | ChIP-Seq | Branson Sonifier 250 | standard | 4 barcoded adapters | gel extraction | after PCR and size selection | GAII |
| Farias-Hesson et al. | *J Biomed Biotechnol.* | 2010 | mitochondrial genes of 32 human samples | Covaris S2 | standard, automated SPRI clean-up | 16 barcoded adapters | Carboxy  Clean up kit | after PCR, before size selection | Solid |
| Lundin et al. | *PLoS ONE* | 2010 | *C. tentans,* lambda phage | nebulizer | standard | 12 barcoded adapters | double SPRI-method | qPCR-assisted pooling | GS FLX Titanium |
| Lennon et al. | *Genome Biology* | 2010 | human genomic DNA | Covaris E210 in 96-well PCR plate | standard, automated SPRI clean-up | 120 barcoded  adapters | double SPRI-method | qPCR-assisted pooling | 454 |
| Meyer & Kircher | *CSH Protocols* | 2010 | 50 targeted human samples | Covaris E210 | blunt end adapter ligation | barcoded primer | none or gel extraction | after PCR, before capture | GAII, HiSeq2000 |
| Adey et al. | *Genome Biology* | 2010 | human exome capture, 96 bacterial genomes from colony | *in vitro* transpositon | *in vitro* transpositon | 96 barcoded adapters or primers | gel extraction, Calliper size selection | after barcoding, no PCR | HiSeq2000 |
| Fisher et al. | *Genome Biology* | 2011 | human exome  capture | Covaris E210 | standard, automated SPRI clean-up with reuse of beads | no barcoding | SPRI cleanup | not applied (1 sample/lane) | “Illumina” |
| Andolfatto et al. | *Genome Research* | 2011 | *Drosophila* (96 well, stainless-steel ball) | restriction enzyme digest | RE ligation | 96 barcoded adapters | gel extraction | before size-selection and PCR | GAII |
| Borgstrom et al. | *PLoS One* | 2011 | 2 spruce samples, 3 human cancer cell line samples | Covaris S2 | standard | no barcoding | double SPRI-method | not applied (1 sample/lane) | HiSeq2000 |
| Rohland & Reich | *Genome Research* | 2012 | 2000 targeted prostate cancer samples | Covaris E210 in 96-well PCR plate | blunt end adapter ligation | 159 barcoded adapters and barcoded primers | double SPRI-method, home made buffer | after 1^st^ PCR, before capture | GAII, HiSeq2000 |
| Our study | *BMC Genomics* | 2012 | 1000 yeast strains  (96 well DNA extraction) | Covaris E210,  Bandelin + PCR tubes | heat-inactivation protocol | 48 barcoded adapters | E-Gel SizeSelect after pooling | before size-selection and PCR | HiSeq2000 |

Innovation
