# Supplementary material for: Genotyping 1000 yeast strains by next-generation sequencing
Source: BMC Genomics. 2013 Feb 9;14:90. doi: 10.1186/1471-2164-14-90 (PMC3575377; doi:10.1186/1471-2164-14-90)
Supplement: Additional file 3 — bmf files (zipped files including + protocol). [file 1471-2164-14-90-S3.zip › biomek-files/PrepEase DNA Extraction from Yeast_96well.doc]

**PrepEase DNA Extraction from Yeast in 96 well plates** [robot program]

1) Inoculate overnight culture in 4 deep well plates with **1000µl** **medium**.
2) Centrifuge plates down at **1000 xg for 2min**, decant supernatant, add 200µl water in first plate, mix, merge cell pellets into a single plate [01] (use filtered 200µl tips), centrifuge at **1000 xg for 2min**, decant supernatant.
3) Add **200 µl of Buffer Y1**, **0.5 μl of Zymolyase** (20 units/µl), and **0.5 µl RNase A** (10mg/ml) per well with multichannel pipet. Pipetmix [02], seal and put on shaker at **25 rpm, at 37°C, for 1 hour**. Add **200µl water**, mix shortly and slowly on vortexer.
4) Centrifuge at **6000 xg** for **5 min**, decant the supernatant (special waste) and put shortly upside down on paper towel.
5) Add **120 µl Homogenization Buffer** (PrepEase) per well with multichannel pipet, pipetmix [02] to re-suspend the pellet completely.
6) Add **100 µl of Chloroform** and **400 µl of Protein Precipitation Buffer** (PrepEase) per well with multichannel pipet to the lysate. Pipetmix [03] and discard tips. Centrifuge at **6000 xg** for **15 min**. [total of 620µl]
7) Transfer **450 µl** of the upper aqueous phase with robot to a new halfdeep well plate containing **340 µl of isopropanol** per well [04]. Put at **-21ºC for 15 min**. Centrifuge at **6000 xg** for **15 min.** [total of 780µl]
8) Decant the supernatant and put upside down on paper towel. Add **500 µl** of **70% ethanol.** Pipetmix [02] and centrifuge at **6000 xg** for **10 min**.
9) Decant the supernatant and put upside down on paper towel. Dry the pellet for **5 min at 37ºC** or **15 min at 20ºC**.
10) Resuspend the DNA pellet in **200 μl** of **DNA Resuspension Buffer or TE,** shake **15 min at 37°C** to dissolve the DNA pellet. Pipettmix [02] and measure the concentration with PicoGreen.

🡪 For centrifuges with max of 3000xg use triple the time compared to centrifugation at 6000xg.

🡪 For shipping, **heat seal** half deep well at 180ºC, 3sec.

**Buffer Y1** (Qiagen’s Yeast Lysis Buffer) store in dark at 4ºC

- 1M sorbitol (182.2 g sorbitol in 600ml water, later adjust to 1L)

- 100mM EDTA (200ml 0.5M Na_2_EDTA, pH 8.0)

- 14mM β-mercaptoethanol (1ml 14.3M β-mercaptoethanol)
